# A deep learning framework for financial time series using stacked autoencoders and long-short term memory

**DOI:** 10.1371/journal.pone.0180944

**Published:** 2017-07-14

**Authors:** Wei Bao, Jun Yue, Yulei Rao

**Affiliations:** 1 Business School, Central South University, Changsha, China; 2 Institute of Remote Sensing and Geographic Information System, Peking University, Beijing, China; University of Rijeka, CROATIA

## Abstract

The application of deep learning approaches to finance has received a great deal of attention from both investors and researchers. This study presents a novel deep learning framework where wavelet transforms (WT), stacked autoencoders (SAEs) and long-short term memory (LSTM) are combined for stock price forecasting. The SAEs for hierarchically extracted deep features is introduced into stock price forecasting for the first time. The deep learning framework comprises three stages. First, the stock price time series is decomposed by WT to eliminate noise. Second, SAEs is applied to generate deep high-level features for predicting the stock price. Third, high-level denoising features are fed into LSTM to forecast the next day’s closing price. Six market indices and their corresponding index futures are chosen to examine the performance of the proposed model. Results show that the proposed model outperforms other similar models in both predictive accuracy and profitability performance.

## Introduction

Stock market prediction is usually considered as one of the most challenging issues among time series predictions [1] due to its noise and volatile features. How to accurately predict stock movement is still an open question with respect to the economic and social organization of modern society. During the past decades, machine learning models, such as Artificial Neural Networks (ANNs) [2] and the Support Vector Regression (SVR) [3], have been widely used to predict financial time series and gain high predictive accuracy [4–8]. In the literature, however, a recent trend in the machine learning and pattern recognition communities considers that a deep nonlinear topology should be applied to time series prediction. An improvement over traditional machine learning models, the new one can successfully model complex real-world data by extracting robust features that capture the relevant information [9] and achieve even better performance than before [10]. Considering the complexity of financial time series, combining deep learning with financial market prediction is regarded as one of the most charming topics [11]. However, this field still remains relatively unexplored.

Generally speaking, there are three main deep learning approaches widely used in studies: convolutional neural networks [12], deep belief networks [13] and stacked autoencoders [14]. The relevant work on deep learning applied to finance has introduced the former two approaches into the research. For example, Ding et al. [15] combine the neural tensor network and the deep convolutional neural network to predict the short-term and long-term influences of events on stock price movements. Also, certain works use deep belief networks in financial market prediction, for example, Yoshihara et al. [16], Shen et al. [17] and Kuremoto et al. [18]. However, regarding whether the stacked autoencoders method could be applied to financial market prediction, few efforts have been made to investigate this issue. Therefore, this paper contributes to this area and provides a novel model based on the stacked autoencoders approach to predict the stock market.

The proposed model in this paper consists of three parts: wavelet transforms (WT), stacked autoencoders (SAEs) and long-short term memory (LSTM). SAEs is the main part of the model and is used to learn the deep features of financial time series in an unsupervised manner. Specifically, it is a neural network consisting of multiple single layer autoencoders in which the output feature of each layer is wired to the inputs of the successive layer. The unsupervised training of SAEs is done one AE at a time by minimizing the error between the output data and the input data. As a result, the SAEs model can successfully learn invariant and abstract features [19].

The other two methods are incorporated to help increase predictive accuracy. LSTM is a type of recurrent neural network (RNN), with feedback links attached to some layers of the network. Unlike conventional RNN, it is well-suited to learn from experience to predict time series when there are time steps with arbitrary size. In addition, it can solve the problem of a vanishing gradient by having the memory unit retain the time related information for an arbitrary amount of time [20]. Evidence has proved that it is more effective than the conventional RNN [21, 22]. Thus, we decide to use this model to predict the stock trends. WT is considered to fix the noise feature of financial time series. It is a widely used technique for filtering and mining single-dimensional signals [23–25]. We use it to denoise the input financial time series and then feed them into the deep learning framework. In summary, the model we introduce in this paper is a combination of the three methods, and we refer to this novel model as WSAEs-LSTM hereafter.

We select six stock indices to test the prediction ability of the proposed model. Those indices include CSI 300 index in A-share market from mainland China, Nifty 50 index representing India stock market, Hang Seng index trading in Hong Kong market, Nikkei 225 index in Tokyo, S&P500 index and DJIA index in New York stock exchange. Technically, we apply WSAEs-LSTM to forecast the movements of each stock index and check how well our model is in predicting stock moving trends.

It is noted that we test the performance of WSAEs-LSTM in several financial markets instead of only one market. This is due to the concern for obtaining robust results. According to the efficient market hypothesis (EMH), the efficiency of a market affects the predictability of its assets. In other words, even though the predictive performances in one market are satisfied, it is still difficult to attribute it to the role of the proposed model. Testing the model in variant market conditions brings us the opportunity to solve the problem and shows us how robust the predictability of our model is. The chosen markets can meet the goal described above. They represent three development stages of financial markets. For example, the stock market in mainland China and India are commonly perceived as developing markets. Though both of them have experienced rapid growth during the past decades, much of their regulation is immature. By contrast, New York stock market is recognized as the most developed market. It is also by far the largest stock exchange and the most efficient market in the world. Besides those above, the stock markets in Hong Kong and Tokyo are in a kind of middle ground between the most developed and the developing state. Thus, our sample setting can help us to examine the validity of our proposed model in different states of the market.

For each stock index, three types of variables are used as model inputs. The first set is historical stock trading data, such as the Open, High, Low and Close price (OHLC) [26–28], and the second is the technical indicators of stock trading. These are commonly used inputs in previous studies [29]. Apart from these, we also introduce the macroeconomic variables as the third type of inputs. As the macro economy can hugely influence stock markets and the advantage of our deep learning model is the ability to extract abstract and invariant features from input variables[30, 31], we believe the addition of macroeconomic variables could improve the model performance.

Regarding the prediction approach, a subsection predictive method described in Chan et al. [32] is applied to get the predicted outcomes of each stock index. Then, we evaluate the model’s performance from two dimensions: predictive accuracy and profitability. The predictive accuracy is evaluated by using three measurements: Mean absolute percentage error (MAPE), correlation coefficient (R) and Theil’s inequality coefficient (Theil U). All of them are widely used indicators to measure whether the predicted value is similar to the actual value [2, 23, 33, 34]. To check the profitability, we establish a buy-and-sell trading strategy [35]. The strategy is applied to obtain the trading returns based on the predicted outcomes from the model. As a benchmark, we also compute the returns of a buy-and-hold strategy for each stock index [32, 36]. The basic idea is that whether the trading returns based on WSAEs-LSTM can outperform the returns of this simple trading strategy, which provides further evidence for the model’s profitability.

To better capture the performance of WSAEs-LSTM, we also introduce other three models and evaluate their predictive accuracy and profitability in forecasting each stock index as the comparisons against our proposed model. The three models include the WLSTM (i.e., a combination of WT and LSTM), LSTM and also the conventional RNN. The former two models are used to check the usefulness of the SAEs method in improving the prediction performance. The last model, RNN, is used as the performance benchmark. As it has been successfully applied to predicting financial time series in previous literature [23, 37, 38], it helps us to get more knowledge regarding how well our proposed model can improve performance compared with the conventional neural network.

All the sample data of this study are collected from WIND database provided by Shanghai Wind Information Co., Ltd, CSMAR database provided by Shenzhen GTA Education Tech. Ltd and a global financial portal: Investing.com. It consists of around 8 years of data from Jul. 2008 to Sep. 2016. Our results show that WSAEs-LSTM outperforms the other three models not only in predictability but also in profitability.

Our work is rooted in a growing research field regarding the application of deep learning method to improve efficiency. For example, deep learning-based methods have dramatically improved the state-of-the-art in image recognition [12, 39–41], speech recognition[42–44], language translation[45, 46] and many other areas such as drug discovery [47] and genomics [48, 49]. The main contribution of this work is that it is the first attempt to apply stacked autoencoders to generate the deep features of the OHLC, technical indicators and macroeconomic conditions as a multivariate signal in order to feed to a LSTM to forecast future stock prices. The proposed deep learning framework, WSAEs-LSTM, can extract more abstract and invariant features compared with the traditional long-short term memory and recurrent neural networks (RNN) approaches.

The rest of this paper is organized into five sections. Section 2 presents the proposed hybrid models with an introduction to multivariate denoising using wavelet, SAEs and LSTM. Section 3 is a description of the inputs and data resource. Section 4 presents the details regarding our experiment design. Section 5 summarizes the observed results and the final section concludes our study.

## Methodology

**Fig 1:**
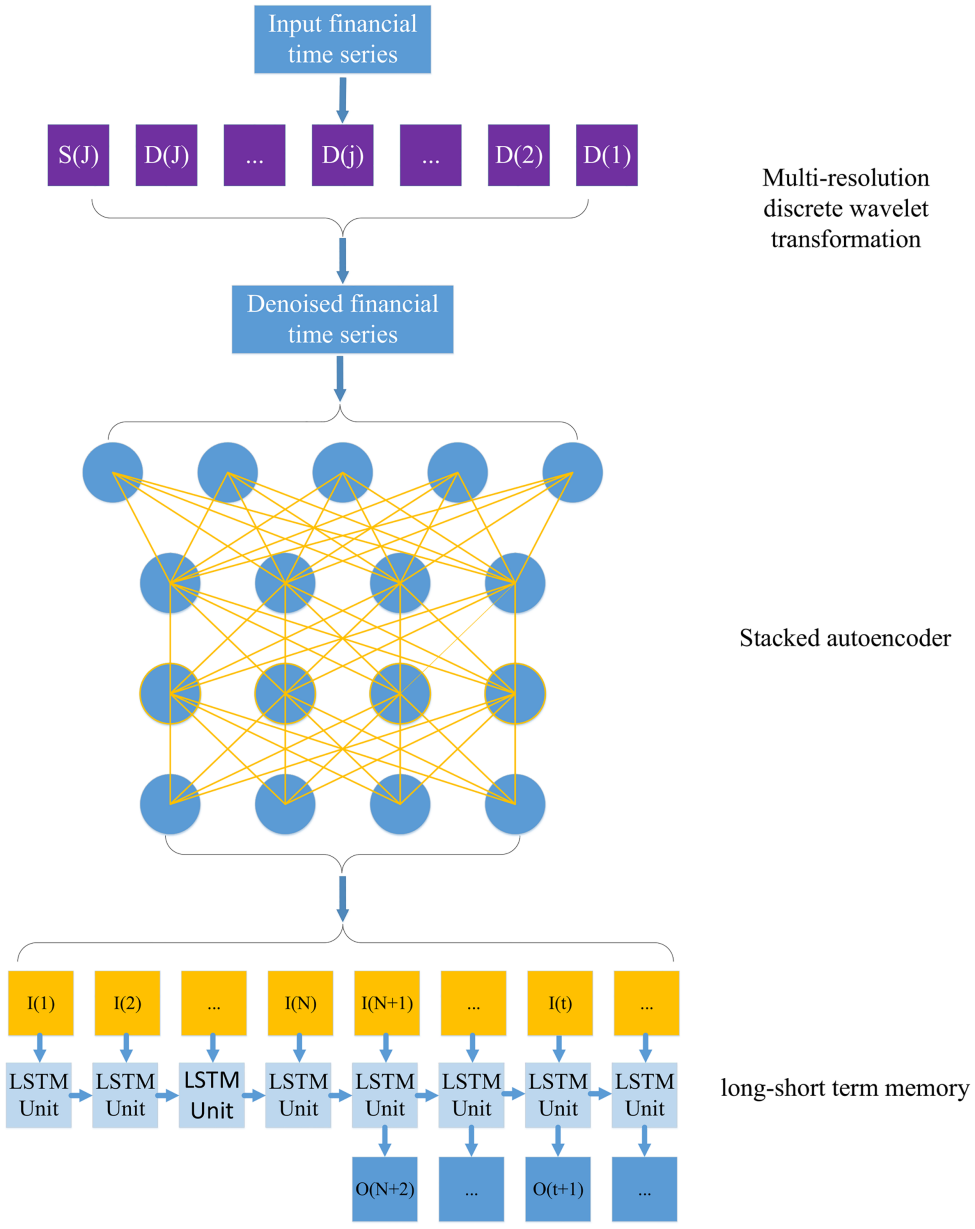
The flowchart of the proposed deep learning framework for financial time series. D(j) is the detailed signal at the j-level. S(J) is the coarsest signal at level J. I(t) and O(t) denote the denoised feature and the one-step-ahead output at time step t, respectively. N is the number of delays of LSTM.

To generate the deep and invariant features for one-step-ahead stock price prediction, this work presents a deep learning framework for financial time series using a deep learning-based forecasting scheme that integrates the architecture of stacked autoencoders and long-short term memory. Fig 1 shows the flow chart of this framework. The framework involves three stages:(1) data preprocessing using the wavelet transform, which is applied to decompose the stock price time series to eliminate noise; (2) application of the stacked autoencoders, which has a deep architecture trained in an unsupervised manner; and (3) the use of long-short term memory with delays to generate the one-step-ahead output. The detailed approach of each block is further detailed as follows.

### Wavelet transform

Wavelet transform is applied for data denoising in this study since it has the ability to handle the non-stationary financial time series data [50]. The key property of wavelet transform is that it can analyze the frequency components of financial time series with time simultaneously compared with the Fourier transform. Consequently, wavelet is useful in handling highly irregular financial time series [51].

This study applies the Haar function as the wavelet basis function because it can not only decompose the financial time series into time and frequency domain but also reduce the processing time significantly [23]. The wavelet transform with the Haar function as a basis has a time complexity of *O*(*n*) with *n* denoting the size of the time series [52].

For continuous wavelet transform (CWT), the wavelet function can be defined by:

$$\phi_{a,\tau }\left(t\right)=\frac{1}{\sqrt{a}}\phi \left(\frac{t-\tau }{a}\right)$$ (1)

where *a* and *τ* are the scale factor and translation factor, respectively. *ϕ*(*t*) is the basis wavelet, which obeys a rule named the wavelet admissibility condition [53]:

$$C_{\phi }=\int_{0}^{\infty }\frac{\left|\Phi \left(\omega \right)\right|}{\omega }d\omega <\infty$$ (2)

where *ϕ*(*ω*) is a function of frequency *ω* and also the Fourier transform of *ϕ*(*t*). Let *x*(*t*) denote a square-integrable function (*x*(*t*) ϵ *L*^2^(*R*)); then CWT with the wavelet *ϕ* can be defined as:

$$CWT_{x}\left(a,\tau \right)=\frac{1}{\sqrt{a}}\int_{-\infty }^{+\infty }x\left(t\right)\bar{\phi \left(\frac{t-\tau }{a}\right)}dt$$ (3)

where $\bar{\phi \left(t\right)}$ denotes its complex conjugate function. The inverse transform of the continuous wavelet transform can be denoted as:

$$x\left(t\right)=\frac{1}{C_{\phi }}\int_{0}^{+\infty }\frac{da}{a^{2}}\int_{-\infty }^{+\infty }CWT_{x}\left(a,\tau \right)\phi_{a,\tau }\left(t\right)d\tau$$ (4)

The coefficients of the continuous wavelet transform have a significant amount of redundant information. Therefore, it is reasonable to sample the coefficients in order to reduce redundancy. Decomposing time series into an orthogonal set of components results in discrete wavelet transform (DWT). Mallat[54] proposed filtering the time series using a pair of high-pass and low-pass filters as an implementation of discrete wavelet transform. There are two types of wavelets, father wavelets *φ*(*t*) and mother wavelets *ψ*(*t*), in the Mallat algorithm. Father wavelets *φ*(*t*) and mother wavelets *ψ*(*t*) integrate to 1 and 0, respectively, which can be formulated as:

∫φ(t)dt=1, ∫ψ(t)dt=0 (5)

The mother wavelets describe high-frequency parts, while the father wavelets describe low-frequency components of a time series. The mother wavelets and the father wavelets in the *j*-level can be formulated as[55]:

$$\varphi_{j,k}\left(t\right)=2^{-\frac{j}{2}}\varphi \left(2^{-j}-k\right)$$ (6)

$$\psi_{j,k}\left(t\right)=2^{-\frac{j}{2}}\psi \left(2^{-j}-k\right)$$ (7)

Financial time series can be reconstructed by a series of projections on the mother and father wavelets with multilevel analysis indexed by *k* ϵ {0,1,2, …} and by *j* ϵ {0,1,2, …*J*}, where *J* denotes the number of multi-resolution scales. The orthogonal wavelet series approximation to a time series *x*(*t*) is formulated by:

$$x\left(t\right)=\underset{k}{\sum }s_{J,k}\varphi_{J,k}\left(t\right)+\underset{k}{\sum }d_{J,k}\psi_{J,k}\left(t\right)+\underset{k}{\sum }d_{J-1,k}\psi_{J-1,k}\left(t\right)+\ldots +\underset{k}{\sum }d_{1,k}\psi_{1,k}\left(t\right)$$ (8)

where the expansion coefficients *s*_*J*,*k*_ and *d*_*J*,*k*_ are given by the projections

$$s_{J,k}=\int \varphi_{J,k}x\left(t\right)dt$$ (9)

$$d_{j,k}=\int \psi_{j,k}x\left(t\right)dt$$ (10)

The multi-scale approximation of time series *x*(*t*) is given as:

$$S_{J}\left(t\right)=\Sigma_{k}s_{J,k}\varphi_{J,k}\left(t\right)$$ (11)

$$D_{j}\left(t\right)=\Sigma_{k}d_{j,k}\psi_{j,k}\left(t\right)$$ (12)

Then, the brief form of orthogonal wavelet series approximation can be denoted by:

$$x\left(t\right)=S_{J}\left(t\right)+D_{J}\left(t\right)+D_{J-1}\left(t\right)+\cdots +D_{1}\left(t\right)$$ (13)

where *S*_*J*_(*t*) is the coarsest approximation of the input time series *x*(*t*). The multi-resolution decomposition of *x*(*t*) is the sequence of {*S*_*J*_(*t*),*D*_*J*_(*t*),*D*_*J*−1_(*t*),…*D*_1_(*t*)}. When the financial time series is very rough, the discrete wavelet transformation can be applied repeatedly by which the risk of overfitting can be reduced. As a result, the two-level wavelet is applied twice in this study for data preprocessing as suggested in [23].

### Stacked autoencoders

Deep learning is a series of models that have the ability to extract deep features from input data with deep neural network architecture. Deep learning models usually have more than three layers. The deep network is typically initialized by unsupervised layer-wise training and then tuned by supervised training with labels that can progressively generate more abstract and high-level features layer by layer [56]. According to recent studies [57, 58], better approximation to nonlinear functions can be generated by deep learning models than those models with a shallow structure.

Several deep neural network architectures have been proposed in recent studies, including deep Boltzmann machines (DBMs) [59], deep belief networks (DBNs) [13] and stacked autoencoders (SAEs) [14]. Restricted Boltzmann machines (RBMs) [60], convolutional neural networks (CNNs) [61], and autoencoders[14] are the frequently used layer-wise training models. In this paper, autoencoders is applied for layer-wise training for the OHLC variables and technical indicators, while SAEs is adopted as the corresponding deep neural network architecture.

**Fig 2:**
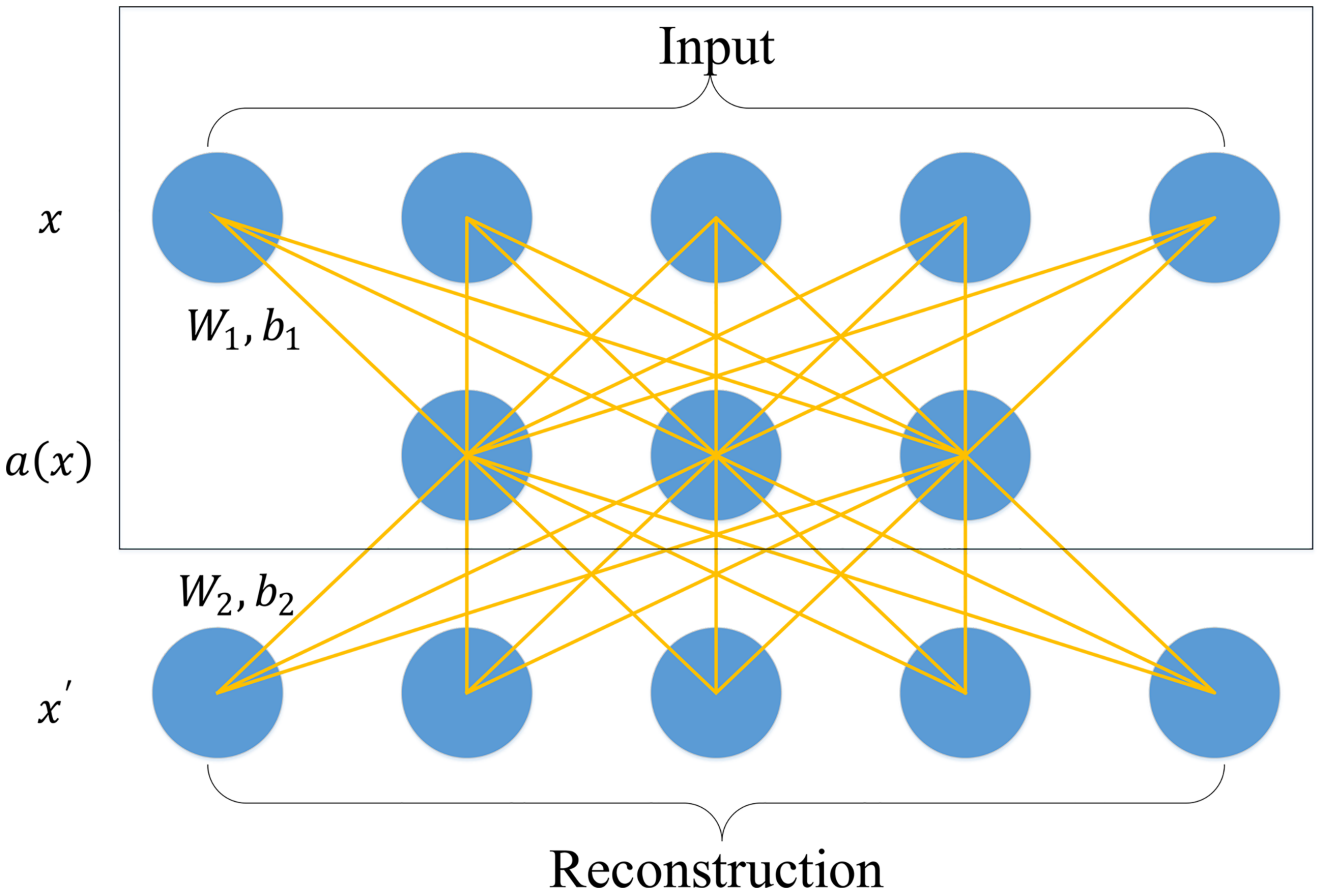
The flowchart of the single layer autoencoder. The model learns a hidden feature *a*(*x*) from input *x* by reconstructing it on *x'*. Here,*W*_1_ and *W*_2_ are the weight of t he hidden layer and the reconstruction layer, respectively. *b*_1_ and *b*_2_ are the bias of the hidden layer and the reconstruction layer, respectively.

Single layer AE is a three-layer neural network; it is illustrated in Fig 2. The first layer and the third layer are the input layer and the reconstruction layer with *k* units, respectively. The second layer is the hidden layer with *n* units, which is designed to generate the deep feature for this single layer AE. The aim of training the single layer AE is to minimize the error between the input vector and the reconstruction vector. The first step of the forward propagation of single layer AE is mapping the input vector to the hidden layer, which is illustrated in the boxed area of Fig 2, while the second step is to reconstruct the input vector by mapping the hidden vector to the reconstruction layer. The two steps can be formulated as:

$$a\left(x\right)=f\left(W_{1}x+b_{1}\right)$$ (14)

$$x^{\prime }=f\left(W_{2}a\left(x\right)+b_{2}\right)$$ (15)

where *x* ϵ R^*k*^ and *x'* ϵ R^*k*^ are the input vector and the reconstructed vector, respectively. *a*(*x*) is the hidden vector generated by the single layer AE. **W**_1_ and **W**_2_ are the weight of the hidden layer and the reconstruction layer, respectively. *b*_1_ and *b*_2_ are the bias of the hidden layer and the reconstruction layer, respectively. *f* is the activate function, which has many alternatives such as sigmoid function, rectified linear unit (ReLU) and hyperbolic tangent. In this paper, *f* is set to be a sigmoid function as in Chen et al. [19]. The optimization function for minimizing the error between the input vector and the reconstruction vector can be formulated as

$${\text{argmin}}_{W_{1},b_{1},W_{2},b_{2}}\left[J\right]=\underset{{}_{W_{1},b_{1},W_{2},b_{2}}}{\text{argmin}}\left[\left(1/2\right)\Sigma_{i=1}^{m}\left\|x_{i}-x_{i}^{\text{'}}\right\|+J_{wd}+J_{sp}\right]$$ (16)

where *J* is the squared reconstruction error of the single layer AE. *x*_*i*_ and $x_{i}^{\text{'}}$ are the *i*th value of the input vector and its corresponding reconstruction vector. *m* is the size of the training dataset, which is the number of trading days in the training stage in this paper. *J*_*wd*_ and *J*_*sp*_ are the weight decay term and the sparse penalty term, which can be formulated as:

$$J_{wd}=\left(1/2\right)\lambda \left({\left\|W_{1}\right\|}_{F}^{2}+{\left\|W_{2}\right\|}_{F}^{2}\right)$$ (17)

$$J_{sp}=\beta \Sigma_{t=1}^{m}KL\left(\rho ∥\hat{\rho_{t}}\right)$$ (18)

where ‖⋅‖_F_ is the Frobenius norm. *λ* and *β* controls the weight decay term and the sparse penalty term. *KL*(⋅) denotes the Kullback-Leibler Divergence. *ρ* is the sparsity parameter, and only a few of the hidden units can be larger than the sparsity parameter. $\hat{\rho_{t}}$ is the average activation of the *t*th hidden layer among the training dataset, which can be formulated as:

$$\hat{\rho_{t}}=\left(1/m\right)\Sigma_{i=1}^{k}a_{t}\left(x_{i}\right)$$ (19)

where *a*_*t*_(*x*_*i*_) denotes the *k*th unit of the *t*th hidden layer among the whole training dataset. The gradient descent algorithm is widely used for solving the optimization problem in SAEs [19, 31]. As a result, the gradient descent algorithm is applied to complete parameter optimization as suggested in Yin et al. [62].

**Fig 3:**
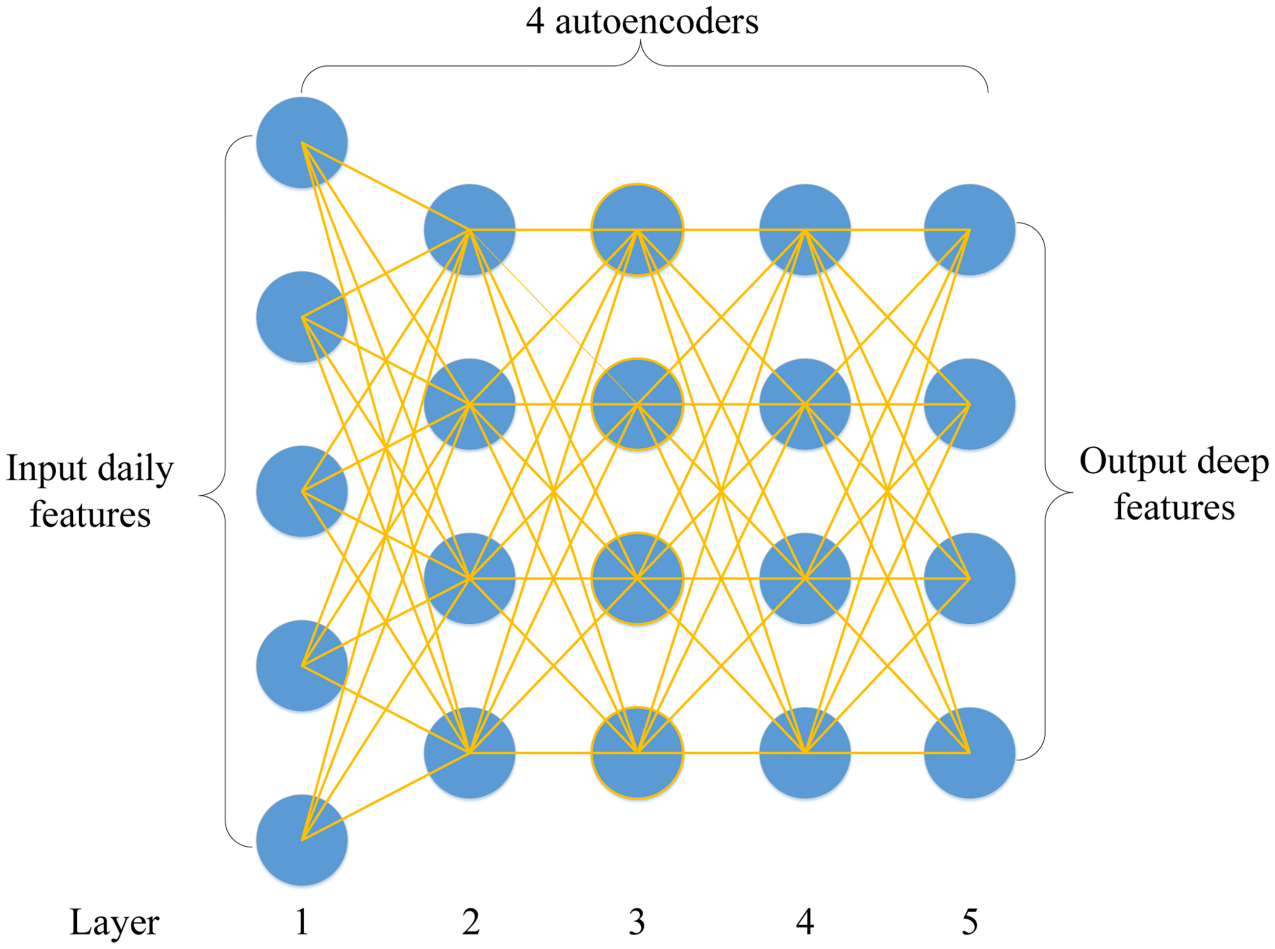
Instance of a stacked autoencoders with 5 layers that is trained by 4 autoencoders.

Stacked autoencoders is constructed by stacking a sequence of single-layer AEs layer by layer [14]. Fig 3 illustrates an instance of an SAE with 5 layers that consists of 4 single-layer autoencoders. The single-layer autoencoder maps the input daily variables into the first hidden vector. After training the first single-layer autoencoder, the reconstruction layer of the first single layer autoencoder is removed, and the hidden layer is reserved as the input layer of the second single-layer autoencoder. Generally speaking, the input layer of the subsequent AE is the hidden layer of the previous AE. Each layer is trained using the same gradient descent algorithm as a single-layer AE by solving the optimization function as formulated in Eq (16) and feeds the hidden vector into the subsequent AE. It is noteworthy that the weights and bias of the reconstruction layer after finishing training each single-layer AE is cast away. In this work, the number of input daily variables for each dataset ranges from 18 to 25; then, the size of hidden layer is set to 10 by trial and error. Depth plays an important role in SAE because it determines qualities like invariance and abstraction of the extracted feature. In this work, the depth of the SAE is set to 5 as recommended in Chen et al. [19].

### Long-short term memory

Long short-term memory is one of the many variations of recurrent neural network (RNN) architecture [20]. In this section, the model of RNN and its LSTM architecture for forecasting the closing price is introduced. We start with the basic recurrent neural network model and then proceed to the LSTM model.

**Fig 4:**
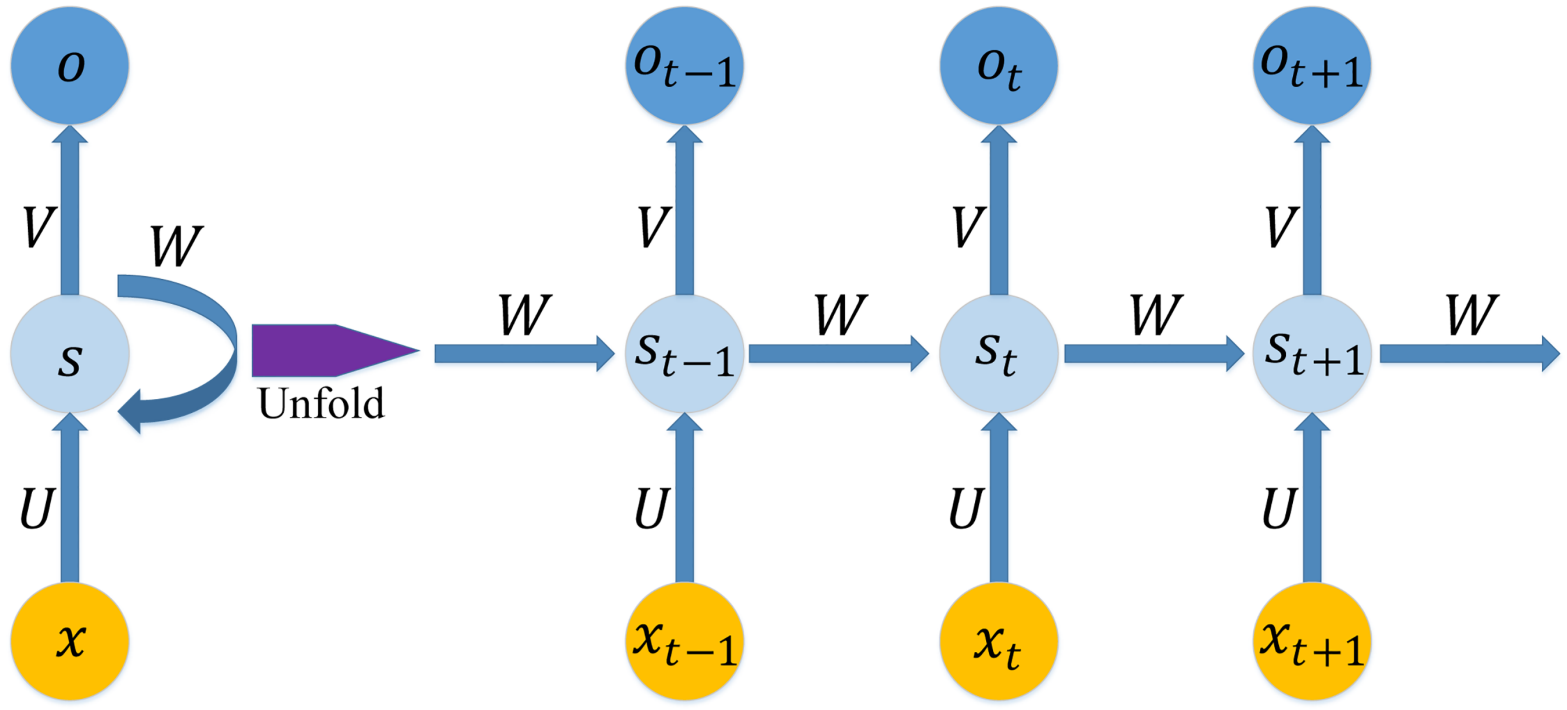
A recurrent neural network and the unfolding architecture. *U*, *V* and *W* are the weights of the hidden layer, the output layer and the hidden state, respectively.*x*_*t*_ and *o*_*t*_ are the input vector and output result at time *t*, respectively.

The RNN is a type of deep neural network architecture [43, 63] that has a deep structure in the temporal dimension. It has been widely used in time series modelling [21, 22, 64–69]. The assumption of a traditional neural network is that all units of the input vectors are independent of each other. As a result, the traditional neural network cannot make use of the sequential information. In contrast, the RNN model adds a hidden state that is generated by the sequential information of a time series, with the output dependent on the hidden state. Fig 4 shows an RNN model being unfolded into a full network. The mathematical symbols in Fig 4 are as follows:

1. *x*_*t*_ is the input vector at time *t*.
2. *s*_*t*_ is the hidden state at time *t*; it is calculated based on the input vector and the previous hidden state. *s*_*t*_ is calculated by:
  $$s_{t}=f\left(Ux_{t}+Ws_{t-1}\right)$$ (20)
  where *f* is the activate function, which has many alternatives such as sigmoid function and ReLU. The initial hidden state *s*_0_ for calculating the first hidden state *s*_1_ is typically initialized to zero.
3. *o*_*t*_ is the output at time *t*, which can be formulated as:
  $$o_{t}=f\left(Vs_{t}\right)$$ (21)
4. *U* and *V* are the weights of the hidden layer and the output layer, respectively. *W* are transition weights of the hidden state.

**Fig 5:**
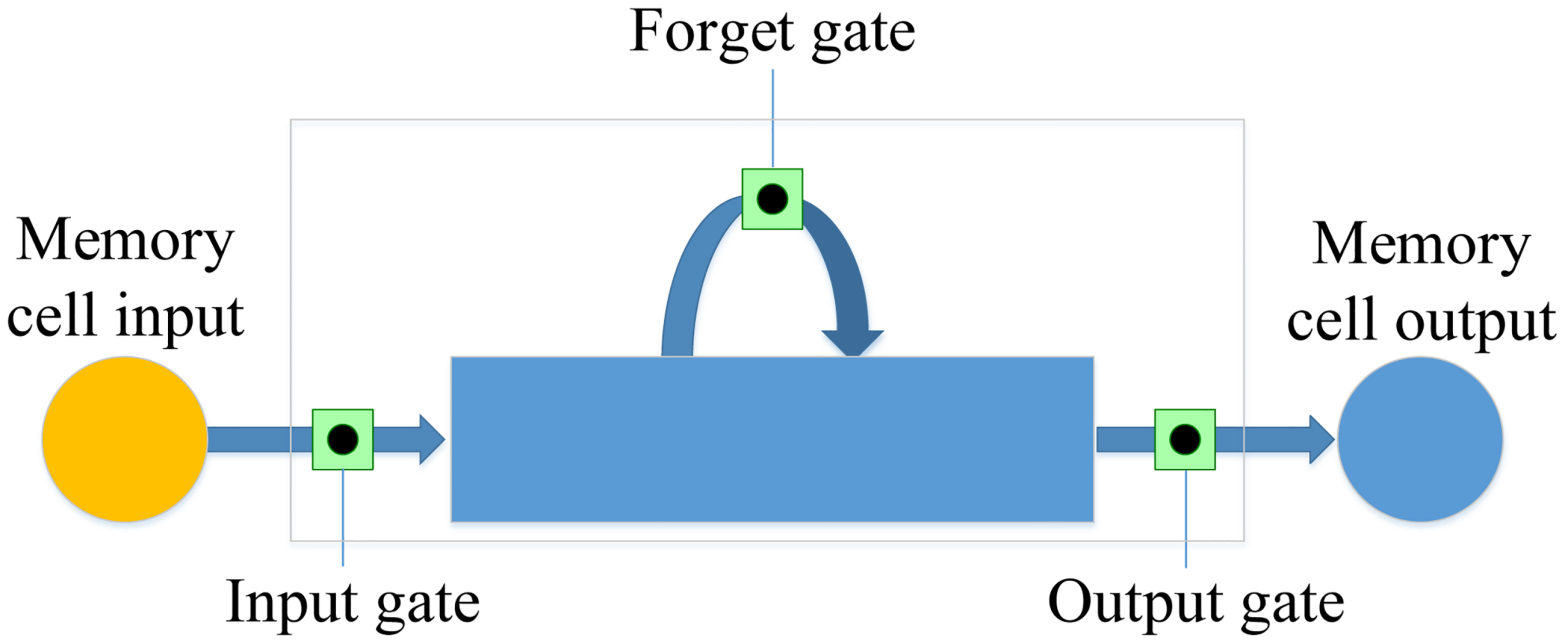
The architecture of an LSTM memory cell.

Although RNN models the time series well, it is hard to learn long-term dependencies because of the vanishing gradient problem [22]. LSTM is an effective solution for combating vanishing gradients by using memory cells [70]. A memory cell is composed of four units: an input gate, an output gate, a forget gate and a self-recurrent neuron, which is illustrated in Fig 5. The gates control the interactions between neighboring memory cells and the memory cell itself. Whether the input signal can alter the state of the memory cell is controlled by the input gate. On the other hand, the output gate can control the state of the memory cell on whether it can alter the state of other memory cell. In addition, the forget gate can choose to remember or forget its previous state.

**Fig 6:**
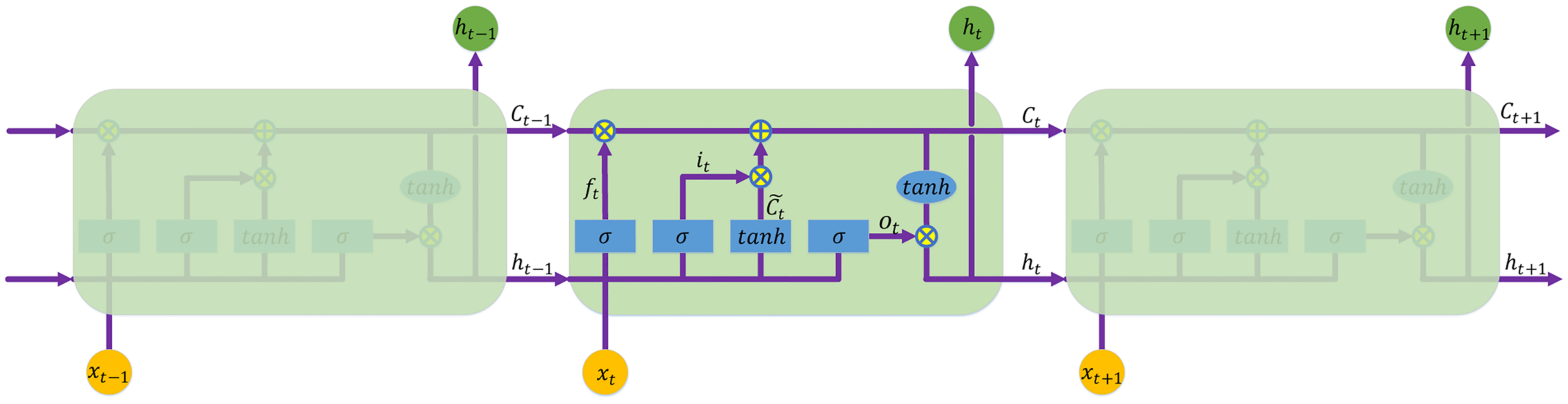
The repeating module in an LSTM. Here,*x*_*t*_ and *h*_*t*_ are the input vector and output result to the memory cell at time *t*, respectively. *h*_*t*_ is the value of the memory cell. *i*_*t*_, *f*_*t*_ and *o*_*t*_ are values of the input gate, the forget gate and the output gate at time *t*, respectively. $\tilde{C_{t}}$ are values of the the candidate state of the memory cell at time *t*.

Fig 6 shows an LSTM model being unrolled into a full network, which describes how the value of each gate is updated. The mathematical symbols in Fig 6 are as follows:

1. *x*_*t*_ is the input vector to the memory cell at time *t*.
2. *W*_*i*_, *W*_*f*_, *W*_*c*_, *W*_*o*_, *U*_*i*_, *U*_*f*_, *U*_*c*_, *U*_*o*_ and *V*_*o*_ are weight matrices.
3. *b*_*i*_, *b*_*f*_, *b*_*c*_ and *b*_*o*_ are bias vectors.
4. *h*_*t*_ is the value of the memory cell at time *t*.
5. *i*_*t*_ and $\tilde{C_{t}}$ are values of the input gate and the candidate state of the memory cell at time *t*, respectively, which can be formulated as:
  $$i_{t}=\sigma \left(W_{i}x_{t}+U_{i}h_{t-1}+b_{i}\right)$$ (22)
  $$\tilde{C_{t}}=tanh\left(W_{c}x_{t}+U_{c}h_{t-1}+b_{c}\right)$$ (23)
6. *f*_*t*_ and *C*_*t*_ are values of the forget gate and the state of the memory cell at time *t*, respectively, which can be calculated by:
  $$f_{t}=\sigma \left(W_{f}x_{t}+U_{f}h_{t-1}+b_{f}\right)$$ (24)
  $$C_{t}=i_{t}*\tilde{C_{t}}+f_{t}*C_{t-1}$$ (25)
7. *o*_*t*_ and *h*_*t*_ are values of the output gate and the value of the memory cell at time *t*, respectively, which can be formulated as:
  $$o_{t}=\sigma \left(W_{o}x_{t}+U_{o}h_{t-1}+V_{o}C_{t}+b_{o}\right)$$ (26)
  $$h_{t}=o_{t}*tanh\left(C_{t}\right)$$ (27)

The architecture of a LSTM network includes the number of hidden layers and the number of delays, which is the number of past data that account for training and testing. Currently, there is no rule of thumb to select the number of delays and hidden layers [21, 22]. In this work, the number of hidden layers and delays are set to 5 and 4 by trial and error. The financial time series is divided into three subsets: training set, validation set, and testing set, with a proportion of 80% training, 10% validation, and 10% testing. The back-propagation algorithm is used to train the WSAEs-LSTM model as well as the models in the experimental control group including WLSTM, LSTM and RNN. The learning rate, batch size and number of epochs are 0.05, 60 and 5000, respectively. The speed of convergence is controlled by the learning rate, which is a decreasing function of time. Setting the number of epochs and the learning rate to 5000 and 0.05 can achieve the convergence of the training. The experimental result will become stable once convergence is achieved though the combinations of parameters are varied [30].

## Data descriptions

In this part, we present details regarding our sample selection and the input variables we choose for model prediction. Also, the data resources are provided in this section.

### Sample selection and input variables

The six stock indices we choose are CSI 300, Nifty 50, Hang Seng index, Nikkei 225, S&P500 and DJIA index. As we noted before, market state may potentially impact the validity of the neural network. Samples from different market conditions can be helpful in solving this problem. The S&P500 and DJIA index are trading in New York stock exchange, which is commonly considered as the most advanced financial market in the world. Therefore, they denote such markets with highest development level. On the contrary, financial markets in both mainland China and India are often classified as new markets. In fact, most of their market institutions are still far from being fully completed. Thus, we choose CSI 300 and Nifty 50 to represent developing markets. In addition to the markets described above, Hang Seng index in Hong Kong and Nikkei 225 index in Tokyo represent a market condition that falls between the developed and developing market. To be honest, financial markets in Hong Kong and Tokyo are usually considered as developed markets in most scenarios. However, in this paper, compared with US stock market, we could say that these two markets are not as mature as US markets. Therefore, those six stock indices give us a natural setting to test the robust of model performances based on different market conditions.

We select three sets of variables as the inputs. Table 1 describes the details. The first set of variables in Panel A is the historical trading data of each index. Following the previous literature, the data includes Open, High, Low, and Close price (OHLC variables) as well as the trading volume. These variables present the basic trading information of each index. Another set of inputs is 12 widely used technical indicators of each index. Panel B gives the details.

**Table 1:** Description of the input variables.

Name	Definition/Implication
**Panel A. Daily Trading Data**	
**Open/Close Price**	nominal daily open/close price
**High/Low Price**	nominal daily highest/lowest price
**Trading volume**	Daily trading volume
**Panel B. Technical Indicator**	
**MACD**	Moving average convergence divergence: displays trend following characteristics and momentum characteristics.
**CCI**	Commodity channel index: helps to find the start and the end of a trend.
**ATR**	Average true range: measures the volatility of price.
**BOLL**	Bollinger Band: provides a relative definition of high and low, which aids in rigorous pattern recognition
**EMA20**	20 day Exponential Moving Average
**MA5/MA10**	5/10 day Moving Average
**MTM6/MTM12**	6/12 month Momentum: helps pinpoint the end of a decline or advance
**ROC**	Price rate of change: shows the speed at which a stock’s price is changing
**SMI**	Stochastic Momentum Index: shows where the close price is relative to the midpoint of the same range.
**WVAD**	Williams's Variable Accumulation/Distribution: measures the buying and selling pressure.
**Panel C. Macroeconomic Variable**	
**Exchange rate**	US dollar Index
**Interest rate**	Interbank Offered Rate

The final set of inputs is the macroeconomic variable. Without a doubt, the macroeconomic conditions across regions also play critical roles in influencing the performance of the stock market. Zhao et al. [71] concludes that the fluctuation of RMB exchange rate can influence the trend of A-share markets in mainland China. Therefore, the addition of macroeconomic variables can be helpful in introducing more information into neural network prediction. We select two kinds of macro variables: the exchange rate and the interest rate. Both rates may affect the money flow in the stock market and then finally impact the performance of stocks. Specifically, we choose US dollar index as the proxy for exchange rate. It is acknowledged that US dollar plays the most important role in the monetary market. Therefore, it alone could be enough to capture the impact from the monetary market to the stock market. Regarding the interest rate, we select the interbank offered rate in each market as the proxy, namely, Shanghai Interbank Offered Rate (SHIBOR), Mumbai Interbank Offered Rate (MIBOR), Hong Kong Interbank Offered Rate (HIBOR), Tokyo Interbank Offered Rate (TIBOR) and Federal funds rate in US.

### Data resource

All of our sample data are from WIND database (http://www.wind.com.cn) provided by Shanghai Wind Information Co., Ltd, CSMAR database (http://www.gtarsc.com) provided by Shenzhen GTA Education Tech. Ltd. and a global financial portal: Investing.com. The sample period in this paper is from 1^st^ Jul. 2008 to 30^th^ Sep. 2016. All of our variables are daily data and can be available on Figshare website (figshare.com/s/acdfb4918c0695405e33, DOI:10.6084/m9.figshare.5028110).

Specifically, it is noted that we do not present the priori time series analysis in this paper. Indeed, the application of classic time series models, such as Auto Regressive Integrated Moving Average (ARIMA), usually requires strict assumptions regarding the distributions and stationarity of time series. As financial time series are usually known to be very complex, non-stationary and very noisy, it is necessary for one to know the properties of the time series before the application of classic time series models [72, 73]. Otherwise, the forecasting effort would be ineffective. However, by using artificial neural networks, a priori analysis of time series is not indispensable. First, ANNs do not require prior knowledge of the time series structure because of their black-box properties [74]. Also, the impact of the stationarity of time series on the prediction power of ANNs is quite small. Related evidence has shown that it is feasible to relax the stationarity condition to non-stationary time series when applying ANNs to predictions [75]. Therefore, we simplify the process for priori data analysis and directly put the data into the model.

## Experiment design

We present the details regarding how we obtain the predicted value and evaluate the performance of each model.

### Prediction approach

**Fig 7:**
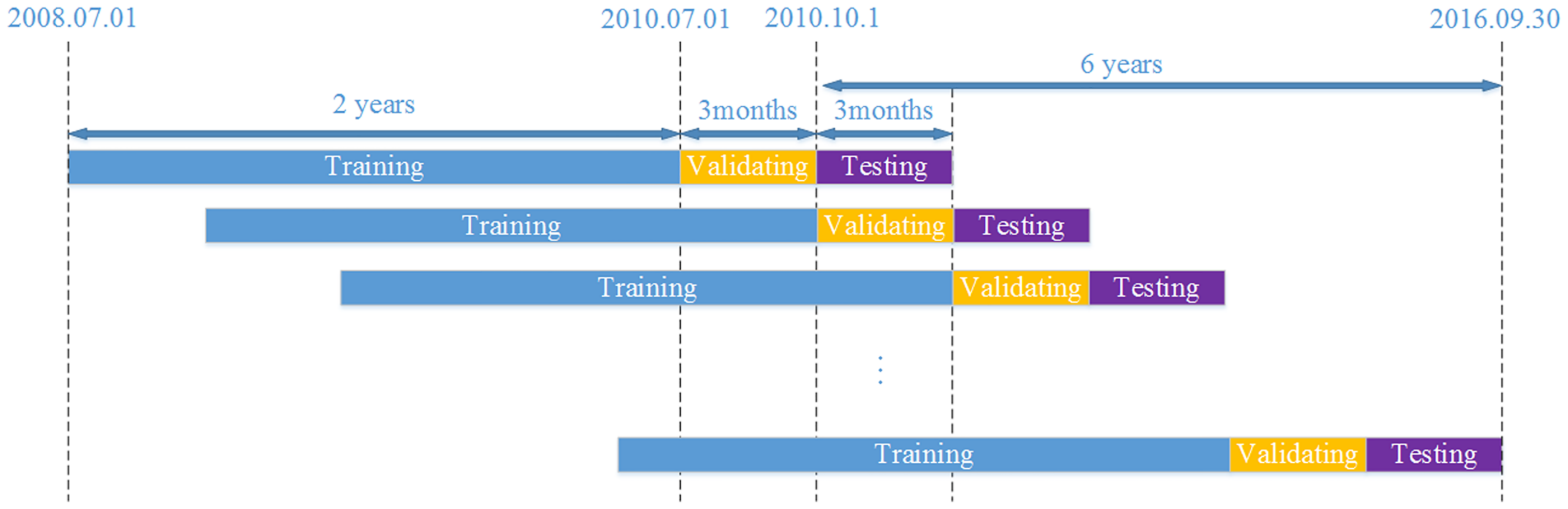
Continuous dataset arrangement for training, validating and testing during the whole sample period.

The prediction procedure follows the subsection prediction method described in Chan et al. [32]. In particular, this procedure consists of three parts. The first part is the training part, which is used to train the model and update model parameters. The second part is the validating part. We use it to tune hyper-parameters and get an optimal model setting. The last one is the test part, where we use the optimal model to predict data. Specifically, as the data are limited, our time frame for each part is inconsistent with that in Chan et al. [32]. In the training part, we use the past two years’ worth of data to train the models. The following period of three months (a calendar quarter) is employed to the validating part. In the test part, in line with popular portfolio management practice, we predict the quarterly performance of each model. This process continues for six years on each quarter from Oct. 2010 to Sep. 2016. Finally, for each stock index, there are 24 quarterly and 6 yearly predicted results. The prediction procedure is illustrated in Fig 7.

To simplify the demonstration of results, we report the yearly performance instead of the quarterly performance in this paper. Thus, the performance of predictive accuracy and trading returns of the models is presented in six-year periods. Details regarding the interval of the six years can be found in Table 2.

**Table 2:** Time interval of the six prediction years.

Year	Time Interval
**Year 1**	2010.10.01–2011.09.30
**Year 2**	2011.10.01–2012.09.30
**Year 3**	2012.10.01–2013.09.30
**Year 4**	2013.10.01–2014.09.30
**Year 5**	2014.10.01–2015.09.30
**Year 6**	2015.10.01–2016.09.30

### Performance measurement

We discuss performance measurements in this part. We first demonstrate the accuracy measurements selected to judge the predictive performance. Next, we argue how we test the profitability performance of each model.

#### Predictive accuracy performance

Previous papers select several indicators to measure how well the model predicts the trend of financial markets [2, 23, 33, 34]. In this paper, we follow their method and choose three classical indicators (i.e., MAPE, R and Theil U) to measure the predictive accuracy of each model. The definitions of these indicators are as follows:

$$MAPE=\frac{\sum_{t=1}^{N}\left|\frac{y_{t}-y_{t}^{*}}{y_{t}}\right|}{N}$$ (28)

$$\;R=\frac{\sum_{t=1}^{N}\left(y_{t}-\bar{y_{t}}\right)\left(y_{t}^{*}-\bar{y_{t}^{*}}\right)}{\sqrt{\sum_{t=1}^{N}{\left(y_{t}-\bar{y_{t}}\right)}^{2}{\left(y_{t}^{*}-\bar{y_{t}^{*}}\right)}^{2}}}$$ (29)

$$\;Theil\;U=\frac{\sqrt{\frac{1}{N}\sum_{t=1}^{N}{\left(y_{t}-y_{t}^{*}\right)}^{2}}}{\sqrt{\frac{1}{N}\sum_{t=1}^{N}{\left(y_{t}\right)}^{2}}+\sqrt{\frac{1}{N}\sum_{t=1}^{N}{\left(y_{t}^{*}\right)}^{2}}}$$ (30)

In these equations, *y*_*t*_ is the actual value and $y_{t}^{*}$ is the predicted value. N represents the prediction period. MAPE measures the size of the error. It is calculated as the relative average of the error. R is a measure of the linear correlation between two variables. Theil U is a relative measure of the difference between two variables. It squares the deviations to give more weight to large errors and to exaggerate errors. If R is bigger, it means that the predicting value is similar to the actual value, while if MAPE and Theil U are smaller, this also indicates that the predicted value is close to the actual value [23, 76].

#### Profitability performance

A buy-and-sell trading strategy is created based on the predicted results of each model. The implication is that under the same trading strategy, we want to find the most valuable model that could earn the highest profits for investors. Actually, the buy-and-sell trading strategy is widely used for profitability performance [35].

The strategy recommends that investors buy when the predicted value of the next period is higher than the current actual value. On the contrary, it recommends that investors sell when the predicted value is smaller than the current actual value. Specifically, the strategy can be described by the following equations:

$$Buy\_signal:\;y_{t+1}^{*}>y_{t}$$ (31)

$$Sell\_signal:\;y_{t+1}^{*}<y_{t}$$ (32)

The *y*_*t*_ denotes the current actual value, and $y_{t+1}^{*}$ is the predicted value for the following time period. The definition of strategy earnings is:

$$R=100\times \left(\sum_{t-1}^{b}\frac{y_{t+1}-y_{t}+\left(y_{t}*B+y_{t+1}*S\right)}{y_{t}}+\sum_{t-1}^{s}\frac{y_{t}-y_{t+1}+\left(y_{t+1}*B+y_{t}*S\right)}{y_{t}}\right)$$ (33)

where R is the strategy returns. b and s denote the total number of days for buying and selling, respectively. B and S are the transaction costs for buying and selling, respectively.

Due to the difficulty in executing the short sale of a basket of stocks in spot markets and the huge transaction costs it produces, we execute this strategy by trading the corresponding index future contracts instead of using stock indices. However, a main concern before this execution is that whether the index futures closely move with their underlying stock indices. In fact, evidence from both theoretical and empirical literature all proves the close connections between stock indices and their corresponding index futures [77–81]. Moreover, to get stronger evidence, we further test the long-term relationships between the six stock indices and their corresponding index futures. Results from Spearman correlation and cointegration test show that all of our indices have a stable long-term relationship with their corresponding index futures (S1 Table). Therefore, we believe our predictive results from spot markets can be successfully applied into their corresponding index future markets.

Based on the above trading rule, we sell short the index future contracts when the predicted price is below the current price and buy the contracts when the predicted price is higher than the current one. We notice that some markets have more than one future product trading in the market. For example, both Hang Seng and S&P 500 index have two types of future products: the standard future contract and the mini future contract. However, unlike the previous two markets, China only has the standard CSI 300 index future. Thus, for the purpose of consistency among markets, we select the standard future product to execute the trading strategy.

To make the results more realistic, we consider the influence of transaction cost on profit. As the cost rates are different among the markets and would be occasionally adjusted for the regulation purpose, we unify the cost rates among our sample markets into one rate within our sample period in order to simplify the calculation procedure. Finally, the chosen cost rate of unilateral trading is 0.01%.

In addition to the buy-and-sell trading strategy, we also incorporate the buy-and-hold trading strategy providing a passive threshold in testing the profitability of proposed models according to previous literature [32, 36]. The trading returns of each model will be compared against the returns of the buy-and-hold strategy. Specifically, as holding the future contract for a long time would be subject to great risk in reality, we execute the buy-and-hold strategy by trading in the spot stock market instead of trading in index future market. The computation procedure of transaction costs in the spot stock market follows the rule that we describe above. Finally, the unified cost in the spot market is 0.25% for buying and 0.45% for selling.

## Results

**Fig 8:**
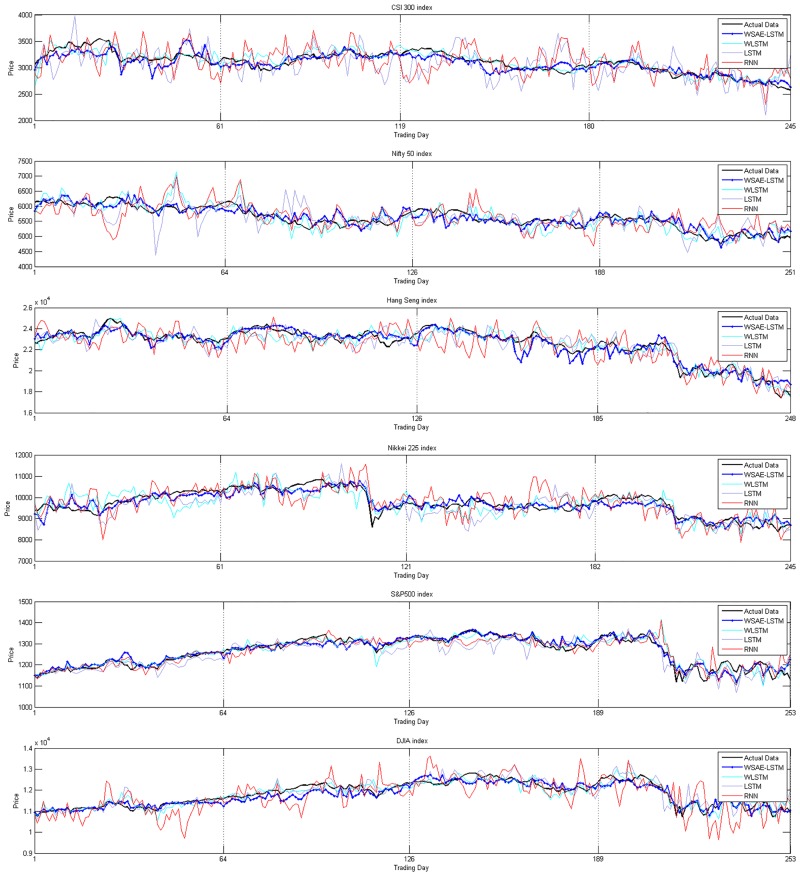
Displays the actual data and the predicted data from the four models for each stock index in Year 1 from 2010.10.01 to 2011.09.30.

For each stock index, we show the yearly predicted data from the four models and the corresponding actual data in the graph. Fig 8 illustrates Year 1 results and the remaining figures for Year 2 to Year 6 can be found in S1–S5 Figs. According to Fig 8 and S1–S5 Figs, we can find that LSTM and RNN have larger variations and distances to the actual data than WSAEs-LSTM and WLSTM. Furthermore, comparing WSAEs-LSTM with WLSTM, the former outperforms the latter: WSAEs-LSTM has less volatility and is closer to the actual trading data than WLSTM. Specifically, the advantage of WSAEs-LSTM in predicting is more obvious in less developed markets than in developed market.

### Predictive accuracy test

The results of predictive accuracy test for each model are reported from Tables 3 to 5. Each table includes the testing results in two stock indices trading in similar market condition. Within each table, each panel demonstrates predictive performance measuring in one of our three accuracy indicators. We separately report the six yearly results and the average value over the six years for each stock index at the same time.

**Table 3:** Predictive accuracy in developing markets.

	CSI 300 index							Nifty 50 index						
Year	Year 1	Year 2	Year 3	Year 4	Year 5	Year 6	Average	Year 1	Year 2	Year 3	Year 4	Year 5	Year 6	Average
**Panel A. MAPE**														
**WSAEs-LSTM**	0.025	0.014	0.016	0.011	0.033	0.016	0.019	0.024	0.019	0.019	0.019	0.018	0.017	0.019
**WLSTM**	0.025	0.029	0.021	0.020	0.038	0.033	0.028	0.034	0.038	0.030	0.025	0.020	0.029	0.029
**LSTM**	0.067	0.077	0.047	0.036	0.053	0.055	0.056	0.043	0.034	0.035	0.035	0.027	0.029	0.034
**RNN**	0.062	0.087	0.052	0.060	0.059	0.075	0.066	0.051	0.038	0.034	0.032	0.036	0.035	0.038
**Panel B. R**														
**WSAEs-LSTM**	0.861	0.959	0.955	0.957	0.975	0.957	0.944	0.878	0.834	0.665	0.972	0.774	0.924	0.841
**WLSTM**	0.841	0.801	0.919	0.864	0.977	0.803	0.868	0.803	0.533	0.312	0.958	0.722	0.844	0.695
**LSTM**	0.440	0.273	0.629	0.742	0.962	0.656	0.617	0.596	0.601	0.027	0.904	0.515	0.772	0.569
**RNN**	0.614	0.363	0.670	0.343	0.943	0.555	0.581	0.506	0.661	0.263	0.929	0.278	0.704	0.557
**Panel C. Theil U**														
**WSAEs-LSTM**	0.017	0.009	0.011	0.007	0.023	0.011	0.013	0.016	0.013	0.013	0.013	0.012	0.012	0.013
**WLSTM**	0.018	0.019	0.014	0.013	0.024	0.022	0.018	0.023	0.025	0.021	0.016	0.014	0.018	0.019
**LSTM**	0.042	0.049	0.030	0.024	0.031	0.036	0.035	0.030	0.023	0.025	0.024	0.018	0.020	0.024
**RNN**	0.044	0.053	0.037	0.041	0.036	0.046	0.043	0.034	0.024	0.024	0.021	0.026	0.023	0.025

**Table 4:** Predictive accuracy in relatively developed markets.

	Hang Seng index							Nikkei 225 index						
Year	Year 1	Year 2	Year 3	Year 4	Year 5	Year 6	Average	Year 1	Year 2	Year 3	Year 4	Year 5	Year 6	Average
**Panel A. MAPE**														
**WSAEs-LSTM**	0.016	0.017	0.012	0.011	0.021	0.013	0.015	0.020	0.016	0.017	0.014	0.016	0.018	0.017
**WLSTM**	0.020	0.027	0.017	0.018	0.028	0.021	0.022	0.033	0.025	0.032	0.025	0.022	0.027	0.028
**LSTM**	0.025	0.027	0.024	0.020	0.023	0.023	0.024	0.036	0.030	0.031	0.029	0.028	0.030	0.031
**RNN**	0.036	0.042	0.030	0.035	0.031	0.032	0.034	0.041	0.039	0.036	0.034	0.034	0.033	0.036
**Panel B. R**														
**WSAEs-LSTM**	0.944	0.924	0.920	0.927	0.904	0.968	0.931	0.895	0.927	0.992	0.885	0.974	0.951	0.937
**WLSTM**	0.935	0.810	0.858	0.833	0.900	0.917	0.876	0.748	0.838	0.973	0.786	0.951	0.906	0.867
**LSTM**	0.895	0.817	0.727	0.812	0.932	0.901	0.847	0.759	0.759	0.972	0.596	0.918	0.881	0.814
**RNN**	0.805	0.679	0.684	0.500	0.870	0.840	0.730	0.694	0.625	0.963	0.575	0.893	0.846	0.766
**Panel C. Theil U**														
**WSAEs-LSTM**	0.011	0.010	0.008	0.007	0.018	0.008	0.011	0.013	0.010	0.010	0.009	0.010	0.011	0.011
**WLSTM**	0.012	0.017	0.011	0.011	0.021	0.013	0.014	0.021	0.017	0.021	0.015	0.013	0.017	0.018
**LSTM**	0.015	0.017	0.016	0.013	0.014	0.015	0.015	0.022	0.022	0.020	0.018	0.018	0.018	0.020
**RNN**	0.022	0.026	0.018	0.021	0.020	0.020	0.021	0.026	0.025	0.023	0.021	0.021	0.021	0.023

**Table 5:** Predictive accuracy in developed markets.

	S&P 500 index							DJIA index						
Year	Year 1	Year 2	Year 3	Year 4	Year 5	Year 6	Average	Year 1	Year 2	Year 3	Year 4	Year 5	Year 6	Average
**Panel A. MAPE**														
**WSAEs-LSTM**	0.012	0.014	0.010	0.008	0.011	0.010	0.011	0.016	0.013	0.009	0.008	0.008	0.010	0.011
**WLSTM**	0.015	0.020	0.012	0.010	0.015	0.015	0.015	0.015	0.018	0.013	0.011	0.017	0.012	0.014
**LSTM**	0.021	0.018	0.013	0.012	0.017	0.022	0.017	0.019	0.026	0.016	0.020	0.020	0.022	0.020
**RNN**	0.017	0.019	0.013	0.019	0.020	0.019	0.018	0.038	0.040	0.029	0.024	0.038	0.026	0.033
**Panel B. R**														
**WSAEs-LSTM**	0.944	0.944	0.984	0.973	0.880	0.953	0.946	0.922	0.928	0.984	0.952	0.953	0.952	0.949
**WLSTM**	0.917	0.886	0.971	0.957	0.772	0.860	0.894	0.915	0.871	0.963	0.911	0.817	0.927	0.901
**LSTM**	0.873	0.905	0.968	0.953	0.795	0.755	0.875	0.860	0.791	0.948	0.751	0.719	0.786	0.809
**RNN**	0.899	0.909	0.966	0.867	0.618	0.822	0.847	0.684	0.579	0.871	0.669	0.256	0.699	0.627
**Panel C. Theil U**														
**WSAEs-LSTM**	0.009	0.010	0.006	0.005	0.008	0.006	0.007	0.010	0.009	0.006	0.005	0.005	0.006	0.007
**WLSTM**	0.011	0.014	0.008	0.007	0.011	0.011	0.010	0.010	0.012	0.008	0.007	0.011	0.008	0.009
**LSTM**	0.014	0.012	0.009	0.007	0.011	0.016	0.011	0.013	0.016	0.010	0.014	0.013	0.014	0.013
**RNN**	0.012	0.012	0.009	0.012	0.013	0.012	0.012	0.025	0.025	0.019	0.016	0.025	0.017	0.021

Table 3 records the model performance in forecasting CSI 300 and Nifty 50. It can be seen from the table that WSAEs-LSTM shows much better performance than the other three models in predicting both stock indices. For example, in predicting CSI 300 index, the average value of MAPE and Theil U of WSAEs-LSTM reach 0.019 and 0.013, respectively, which is much less than those of the other three models. Besides, the indicator R has an average value of 0.944, which is the highest one among the four models. In fact, WSAEs-LSTM outperforms the other three not only on average but also in each year. To confirm the robustness of our findings, we examine the statistical significance of the differences between WSAEs-LSTM and the other three models. Specifically, we compare the 24 quarterly results of WSAEs-LSTM with those of the three models for each accuracy indicators. The statistic approach, T-test, is used for these comparisons. Finally, the statistical evidence proves that the differences between WSAEs-LSTM and the rest three models are all statistically significant at 5% level in both stock indices.

Tables 4 and 5 present the models’ performance in the rest four stock indices: Table 4 demonstrates model performance in Hong Kong and Tokyo markets while Table 5 reports the results in S&P 500 and DJIA index. Similar to what we have found in Table 3, WSAEs-LSTM still has the lowest MAPE and Theil U and the highest R than the other three models not only from the perspective of average value but also from the perspective of yearly results. Still, these differences between our proposed model and the other three models pass the statistical test at 5% significant level. This concludes that WSAEs-LSTM can stably obtain lower prediction errors and higher predictive accuracy than the other three models regardless of market conditions.

Besides the findings described above, we also discover an interesting pattern based on our data: the difference between the predictability of one specific model in forecasting two stock indices is quite small if these two stock indices are traded in markets with similar development state, while the difference would be increased if the two stock indices are traded in markets with different development states. For example, MAPE of WSAEs-LSTM in predicting S&P 500 and DJIA is 0.011, while it increases to 0.019 when predicting CSI 300 and Nifty 50. Similar patterns are also existed for the rest three models. Even though all models exhibit this pattern, the extent of impacts from market condition is different among models. For example, the market condition seems quite influential on RNN. Its average MAPE value ranges from 0.018 to 0.066 among the six stock indices. That means the worst predictability of RNN is only around one-fourth of its best predictability. Both WLSTM and LSTM exhibit similar patterns as RNN. By contrast, the performance of WSAEs-LSTM is quite stable across markets comparing with these three models. This could be due to the fact that SAEs is more powerful in processing noise data than the other three. The implication of these findings is that our model could be more valuable than others in predicting systems that are less mature and have higher volatility.

### Profitability test

The results of profitability test are shown in Table 6. Similarly, we report both yearly returns and the average returns over the six years. Each panel describes the trading returns gained by the models in a specific market condition. In particular, the last row in each panel reports the returns of the buy-and-hold strategy in trading a specific stock index.

**Table 6:** Profitability performance of each model.

Year	Year 1	Year 2	Year 3	Year 4	Year 5	Year 6	Average	Year 1	Year 2	Year 3	Year 4	Year 5	Year 6	Average
**Panel A. Developing market**														
	**CSI 300 Index**							**Nifty 50 Index**						
**WSAEs-LSTM**	46.428	59.580	65.685	71.897	63.951	70.613	63.026	56.911	51.710	66.765	37.107	31.882	28.134	45.418
**WLSTM**	26.766	21.942	36.502	20.304	89.984	42.283	39.630	13.835	19.254	13.679	22.464	44.032	28.436	23.617
**LSTM**	-15.802	-16.802	24.082	1.345	57.903	53.479	17.368	26.247	16.509	0.921	-8.459	37.180	18.279	15.113
**RNN**	-3.310	1.750	8.827	-0.747	47.308	-8.630	7.533	-7.757	-10.558	19.691	22.685	29.492	-7.435	7.686
**Buy-and-hold**	-19.630	-12.595	3.065	0.292	35.619	3.430	1.697	-23.564	13.926	-1.510	30.885	-2.787	5.477	3.738
**Panel B. Relatively developed market**														
	**Hang Seng Index**							**Nikkei 225 Index**						
**WSAEs-LSTM**	75.844	81.890	54.685	49.715	64.727	59.949	64.468	53.463	37.846	81.032	48.832	59.419	76.030	59.437
**WLSTM**	25.697	19.221	37.410	23.149	59.030	51.220	35.955	27.774	10.196	43.143	32.411	31.252	62.569	34.558
**LSTM**	16.381	25.733	21.892	1.545	23.680	62.717	25.325	14.032	31.507	52.324	-15.605	8.774	26.272	19.551
**RNN**	5.287	40.810	-7.114	-17.024	27.845	48.463	16.378	-7.329	-6.967	8.318	-15.389	42.258	50.950	11.974
**Buy-and-hold**	-27.321	16.341	7.472	-2.821	-9.264	9.455	-1.023	-19.371	1.568	49.339	9.790	6.365	-6.728	6.827
**Panel C. Developed market**														
	**S&P 500 Index**							**DJIA Index**						
**WSAEs-LSTM**	71.316	48.351	39.239	9.940	61.187	45.950	45.997	76.742	55.433	41.700	47.034	82.503	80.522	63.989
**WLSTM**	40.246	25.726	21.964	15.906	31.839	18.168	25.641	47.180	35.174	30.682	29.532	30.605	54.885	38.009
**LSTM**	-7.633	23.138	20.710	-2.678	2.010	34.254	11.633	33.868	22.951	17.919	-5.952	4.493	37.578	18.476
**RNN**	18.319	11.930	11.261	-8.475	12.266	3.607	8.152	4.787	6.763	-2.094	8.730	37.368	17.814	12.228
**Buy-and-hold**	-12.271	22.755	13.212	13.747	-5.358	9.819	6.984	-7.860	19.070	9.725	9.620	-7.144	9.356	5.461

Panel A demonstrates the profitability performance of each model in developing markets. The left part is the trading performance based on predicted data from CSI 300, while the right part is trading performance based on predicted data from Nifty 50. The results suggest that WSAEs-LSTM earns substantially more profits than the other three models. For example, the average annual earnings of the proposed model can reach up to 63.026% in mainland China and 45.418% in India market, while the annual earnings of the other three models are nearly all below 40%. Regarding each yearly returns, WSAEs-LSTM also outperforms the other models. It can almost stably gain more than 40% earnings in every year, which is really difficult for the other three models.

Panel B and C reports the trading returns in relatively developed and developed markets, respectively. Similar as the findings in Panel A, WSAEs-LSTM can acquire stable earnings in every year, while other models face larger variance in trading earnings. In addition, from the perspective of average earnings within our sample period, our proposed model still earns the highest profits according to the results in Panel B and C.

Furthermore, to achieve a robust conclusion, we also test whether the returns differences between WSAEs-LSTM and the remaining three models are statistically significant. Again, we compare the 24 quarterly returns among the models. The t-test results show that our return differences between WSAEs-LSTM and the other three models all pass the significant test at the 5% level. Therefore, our findings support that WSAEs-LSTM has the best predictability among the four models.

## Conclusion

This paper builds a novel forecasting framework to predict the one-step-ahead closing price of six popular stock indices traded in different financial markets. The procedure for building this forecasting framework is as follows: First, the denoised time series is generated via discrete wavelet transform using the Haar wavelet; second, the deep daily features are extracted via SAEs in an unsupervised manner; third, long-short term memory is used to generate the one-step-ahead output in a supervised manner. Our input variables include the daily OHLC variables, technical indicators and macroeconomic variables. The main contribution of this work to the community is that it is the first attempt to introduce SAEs method to extract deep invariant daily features of financial time series. In addition, the deep learning framework is proposed with a complete set of modules for denoising, deep feature extracting instead of feature selection and financial time series fitting. Within this framework, the forecasting model can be developed by replacing each module with a state-of-the-art method in the areas of denoising, deep feature extracting or time series fitting.

We test the predictive accuracy and profitability of our proposed model compared with the other three models. The results provide evidence that it can outperform the other three in both predictive accuracy and profitability regardless of which stock index is chosen for examination. Although the proposed integrated system has a satisfactory predictive performance, it still has some insufficiencies. For example, a more advanced hyper-parameters selection scheme might be embedded in the system to further optimize the proposed deep learning framework. In addition, deep learning methods are time-consuming, and more attention needs to be paid to GPU-based and heterogeneous computing-based deep learning methods. All of these could be enhanced by future studies.

## Supporting information

S1 Fig Displays the actual data and the predicted data from the four models for each stock index in Year 2 from 2011.10.01 to 2012.09.30. (TIF)

S2 Fig Displays the actual data and the predicted data from the four models for each stock index in Year 3 from 2012.10.01 to 2013.09.30. (TIF)

S3 Fig Displays the actual data and the predicted data from the four models for each stock index in Year 4 from 2013.10.01 to 2014.09.30. (TIF)

S4 Fig Displays the actual data and the predicted data from the four models for each stock index in Year 5 from 2014.10.01 to 2015.09.30. (TIF)

S5 Fig Displays the actual data and the predicted data from the four models for each stock index in Year 6 from 2015.10.01 to 2016.09.30. (TIF)

S1 Table Relationship between indices and corresponding index futures. (PDF)
