# Supplementary figures and images for: A deep learning framework for financial time series using stacked autoencoders and long-short term memory
Source: PLoS One. 2017 Jul 14;12(7):e0180944. doi: 10.1371/journal.pone.0180944 (PMC5510866; doi:10.1371/journal.pone.0180944)

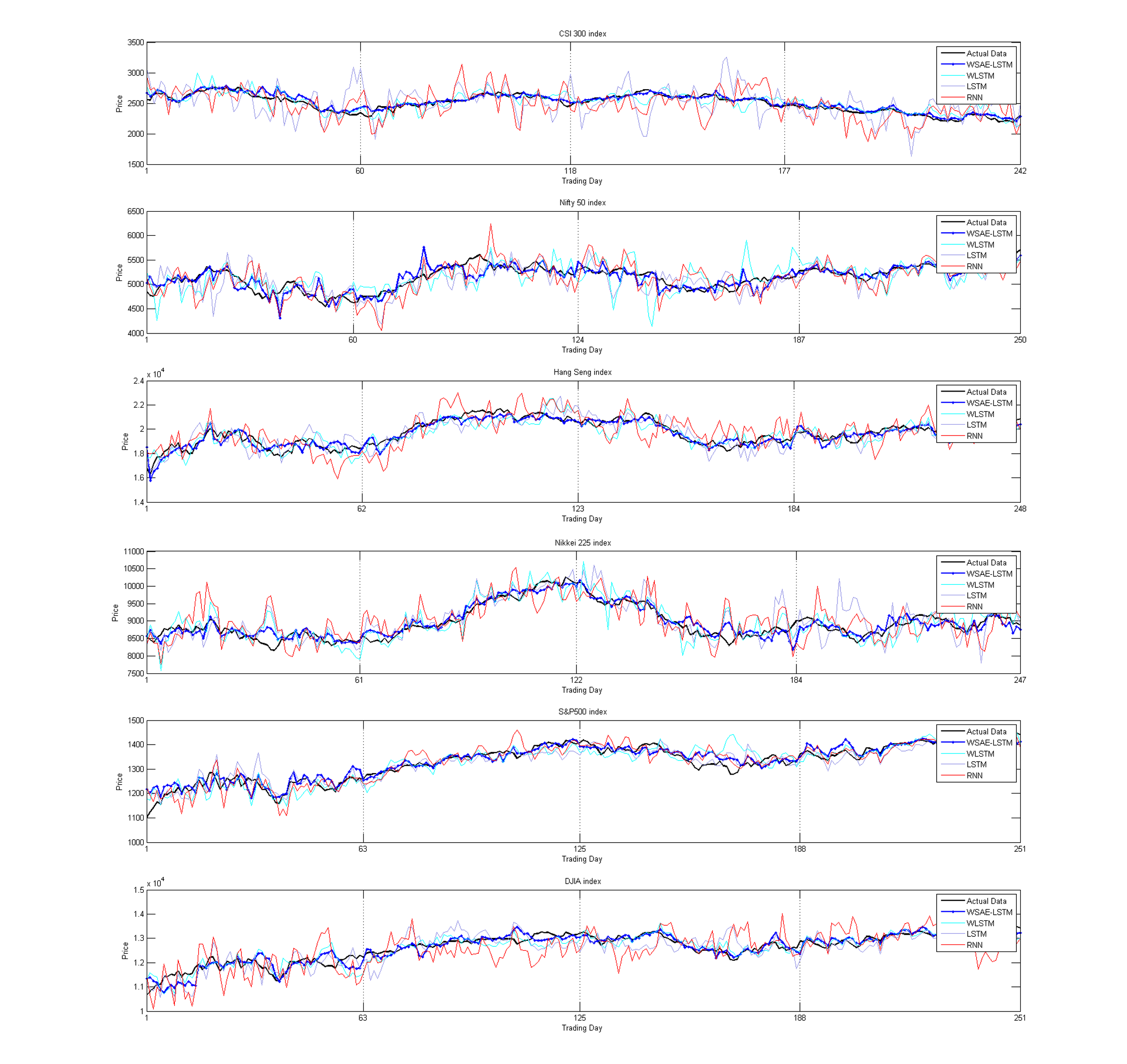

Supplement: S1 Fig — (TIF) [file pone.0180944.s001.tif]

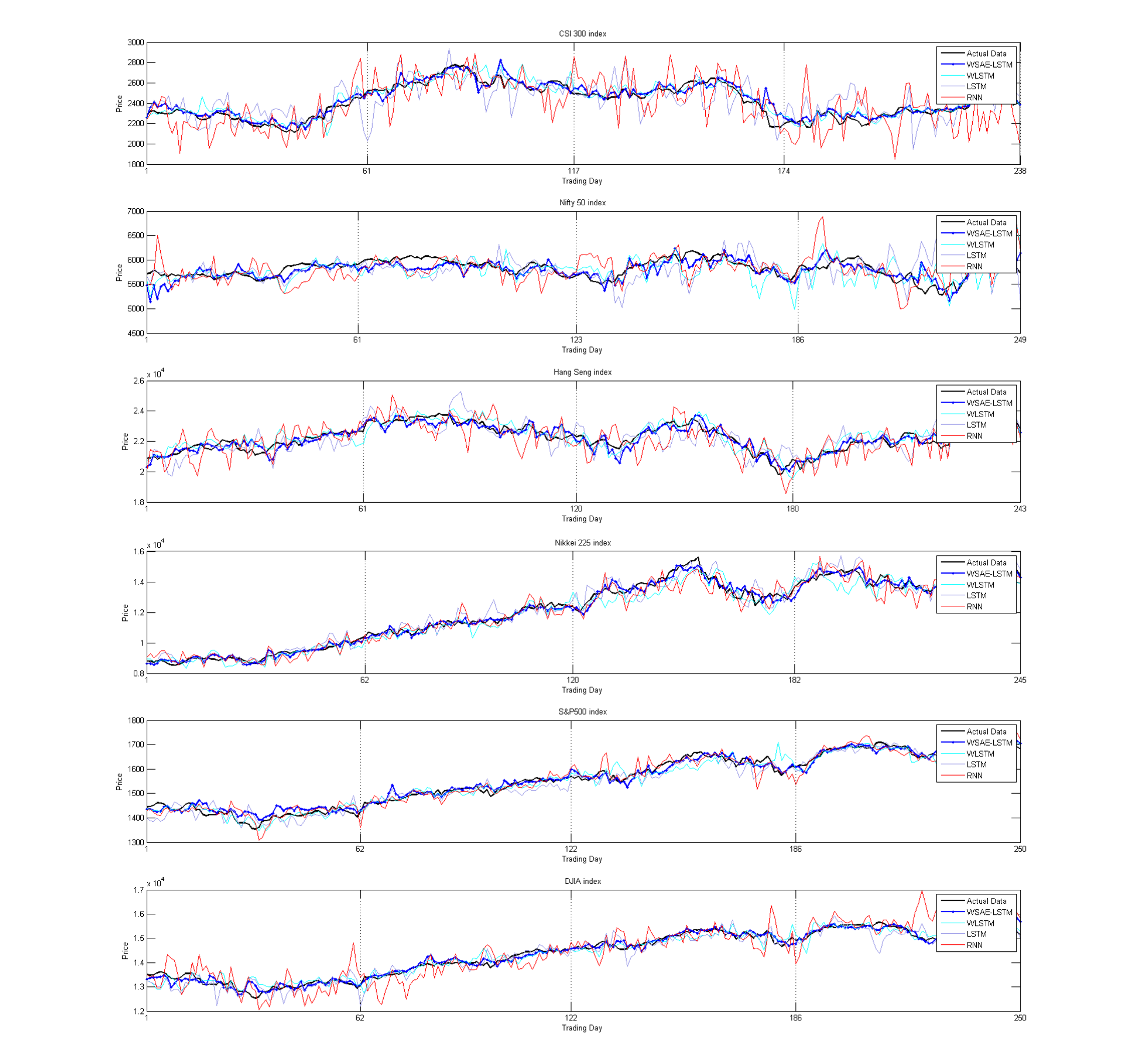

Supplement: S2 Fig — (TIF) [file pone.0180944.s002.tif]

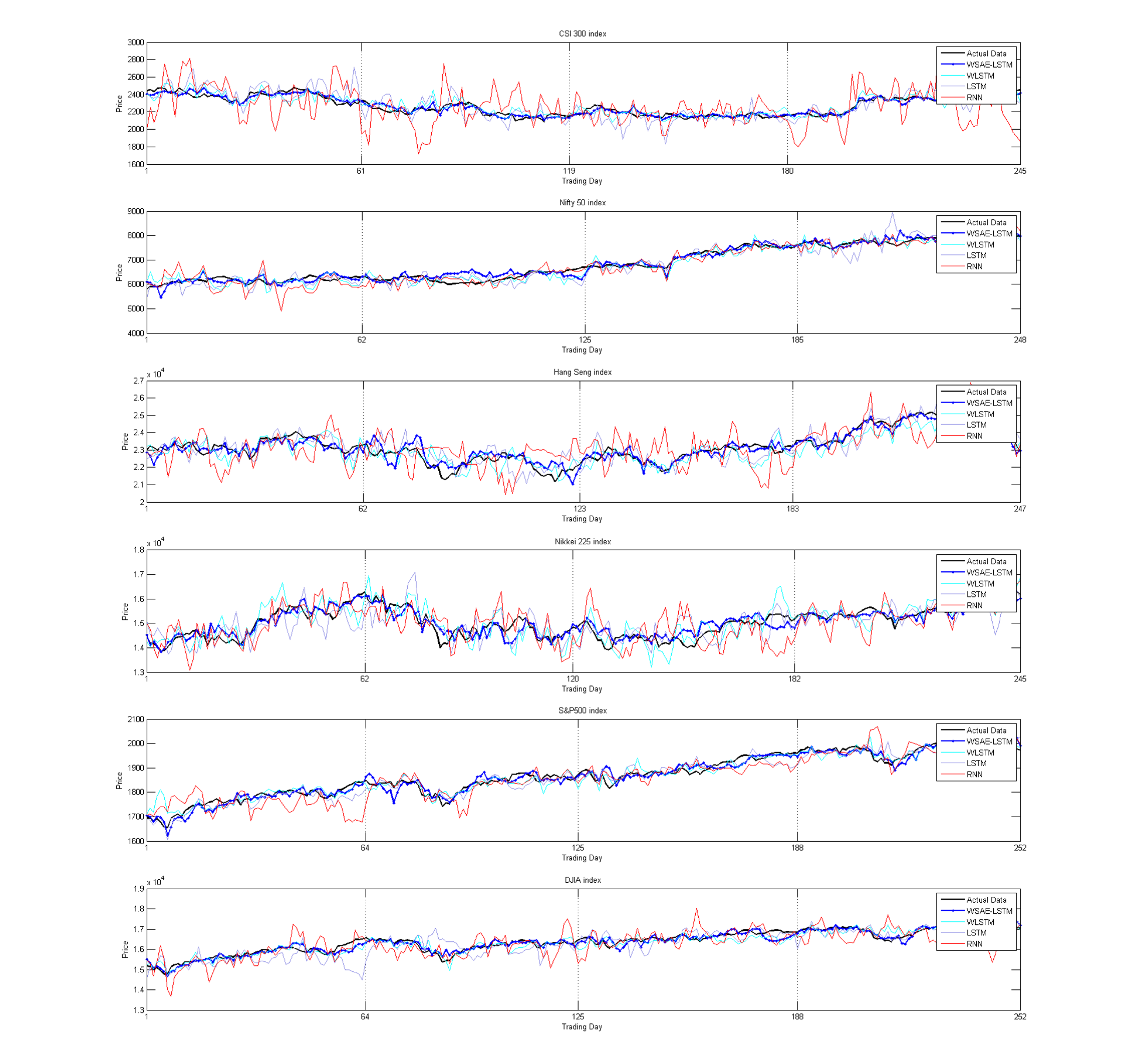

Supplement: S3 Fig — (TIF) [file pone.0180944.s003.tif]

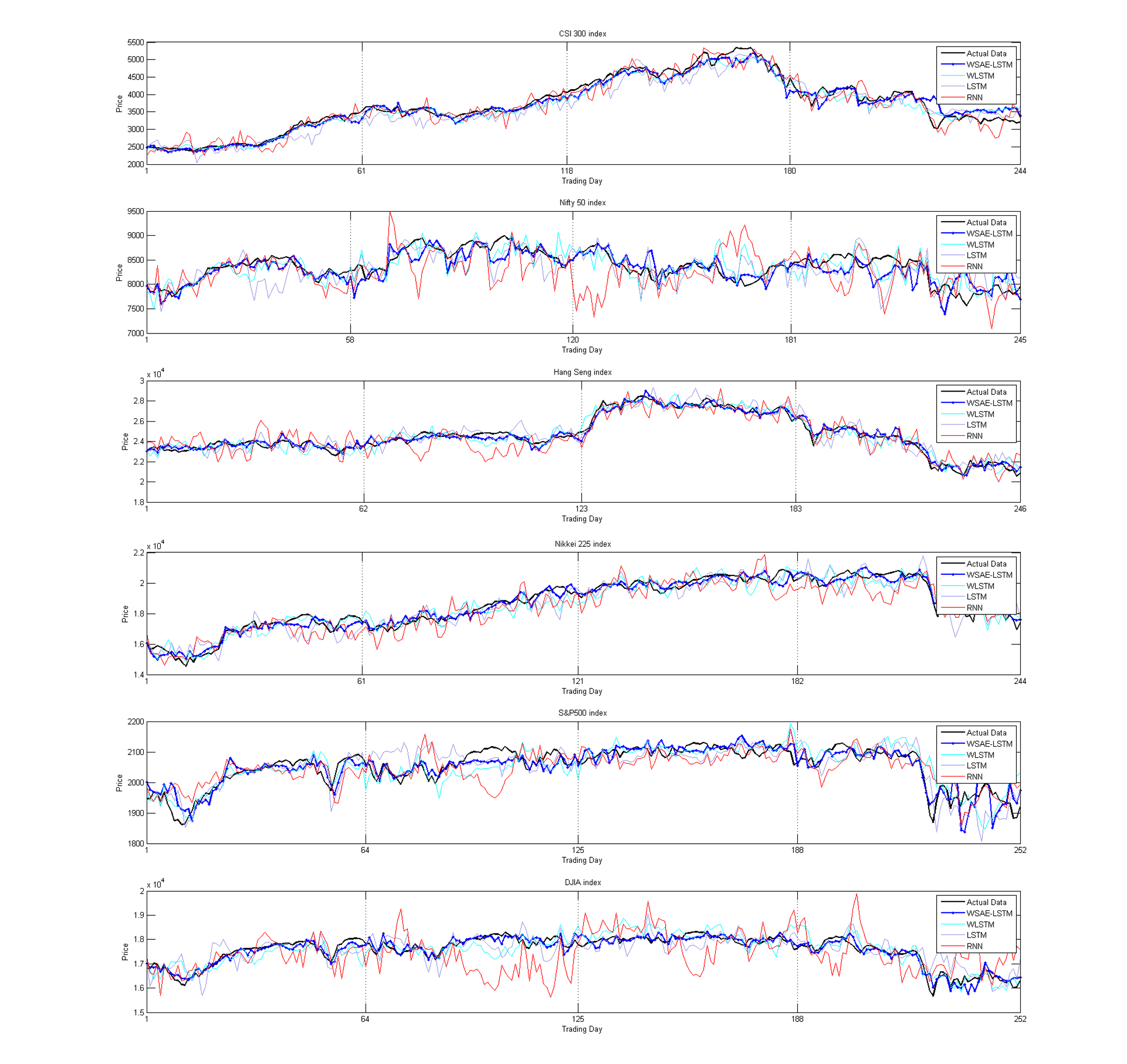

Supplement: S4 Fig — (TIF) [file pone.0180944.s004.tif]

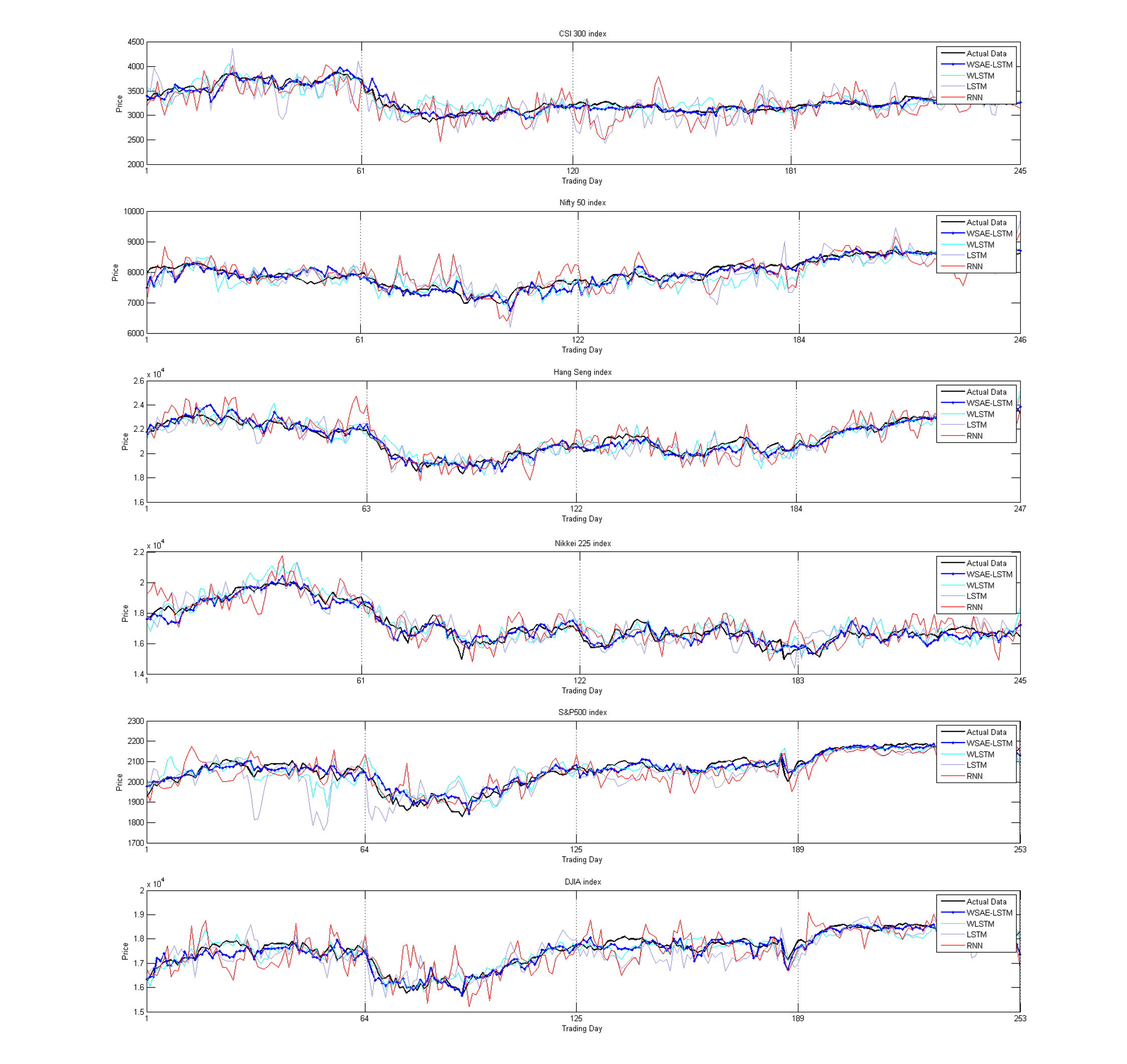

Supplement: S5 Fig — (TIF) [file pone.0180944.s005.tif]
