# Supplementary material for: A deep learning framework for financial time series using stacked autoencoders and long-short term memory
Source: PLoS One. 2017 Jul 14;12(7):e0180944. doi: 10.1371/journal.pone.0180944 (PMC5510866; doi:10.1371/journal.pone.0180944)
Supplement: S1 Table — (PDF) [file pone.0180944.s006.pdf]

S1 Table. Relationship between indices and corresponding index futures

Index	Spearman Correlation	Johansen Cointegration	
		Trace Statistic	Max-Eigen Statistic
S&P 500	0.999859***	106.0300***	104.5311***
DJIA	0.999734***	73.75235***	71.61849***
Hang Seng	0.999053***	120.4101***	113.6928***
Nikkei 225	0.999874***	125.5730***	124.5978***
CSI 300	0.997851***	60.19136***	57.53962***
Nifty 50	0.999809***	90.31696***	89.99845***

***Significant at 1% level
